# DNA replication stress restricts ribosomal DNA copy number

**DOI:** 10.1371/journal.pgen.1007006

**Published:** 2017-09-15

**Authors:** Devika Salim, William D. Bradford, Amy Freeland, Gillian Cady, Jianmin Wang, Steven C. Pruitt, Jennifer L. Gerton

**Affiliations:** 1 Stowers Institute for Medical Research, Kansas City, MO, United States of America; 2 Open University, Milton Keynes MK7 6BJ, United Kingdom; 3 Department of Molecular and Cellular Biology, Roswell Park Cancer Institute, Buffalo, NY, United States of America; 4 Department of Biostatistics and Bioinformatics, Roswell Park Cancer Institute, Buffalo, NY, United States of America; 5 Department of Biochemistry and Molecular Biology, University of Kansas Medical Center, Kansas City, KS, United States of America; Ohio State University, UNITED STATES

## Abstract

Ribosomal RNAs (rRNAs) in budding yeast are encoded by ~100–200 repeats of a 9.1kb sequence arranged in tandem on chromosome XII, the ribosomal DNA (rDNA) locus. Copy number of rDNA repeat units in eukaryotic cells is maintained far in excess of the requirement for ribosome biogenesis. Despite the importance of the repeats for both ribosomal and non-ribosomal functions, it is currently not known how “normal” copy number is determined or maintained. To identify essential genes involved in the maintenance of rDNA copy number, we developed a droplet digital PCR based assay to measure rDNA copy number in yeast and used it to screen a yeast conditional temperature-sensitive mutant collection of essential genes. Our screen revealed that low rDNA copy number is associated with compromised DNA replication. Further, subculturing yeast under two separate conditions of DNA replication stress selected for a contraction of the rDNA array independent of the replication fork blocking protein, Fob1. Interestingly, cells with a contracted array grew better than their counterparts with normal copy number under conditions of DNA replication stress. Our data indicate that DNA replication stresses select for a smaller rDNA array. We speculate that this liberates scarce replication factors for use by the rest of the genome, which in turn helps cells complete DNA replication and continue to propagate. Interestingly, tumors from mini chromosome maintenance 2 (MCM2)-deficient mice also show a loss of rDNA repeats. Our data suggest that a reduction in rDNA copy number may indicate a history of DNA replication stress, and that rDNA array size could serve as a diagnostic marker for replication stress. Taken together, these data begin to suggest the selective pressures that combine to yield a “normal” rDNA copy number.

## Author summary

Eukaryotic genomes contain many copies of ribosomal DNA (rDNA) genes, usually far in excess of the requirement for cellular ribosome biogenesis. rDNA array size is highly variable, both within and across species. Although it is becoming increasingly evident that the rDNA locus serves extra-coding functions, and several pathways that contribute to maintenance of normal rDNA copy number have been discovered, the mechanisms that determine optimal rDNA array size in a cell remain unknown. Here we identify DNA replication stress as one factor that restricts rDNA copy number. We present evidence suggesting that DNA replication stress selects for cells with smaller rDNA arrays, and that contraction of the rDNA array provides a selective advantage to cells under conditions of DNA replication stress. Loss of rDNA copies may be a useful indicator of a history of replication stress, as observed in a mouse model for cancer.

## Introduction

Ribosomal RNAs (rRNAs) in budding yeast are encoded by ~100–200 repeats of a 9.1kb sequence arranged in tandem on the long arm of chromosome XII, the ribosomal DNA (rDNA) locus. Each 9.1kb unit is composed of a region encoding a pre-35S rRNA that gives rise to 25S, 18S and 5.8S rRNAs (transcribed by RNA polymerase I), a small 5S rRNA (transcribed by RNA polymerase III), and two intergenic spacers (IGS1 and IGS2). IGS2 contains an rDNA origin of replication, rARS, and a cohesin associating sequence (CAR). IGS1 contains a replication fork barrier site (RFB) to which Fob1 binds; the binding of Fob1 inhibits DNA replication in the direction opposite to 35S rDNA transcription, preventing the head on collision of transcription and replication machinery [1]. IGS1 also contains a bi-directional RNA polymerase II promoter, E-pro, whose activity is normally suppressed by the binding of Sir2, an NAD-dependent histone deacetylase [2].

Two major features of the rDNA locus give it the unique potential to sense the environment and tune cellular response–high instability, and the wide range of copy number variation it can accommodate. Both features have been extensively studied, particularly in budding yeast, however molecular mechanisms of regulation of instability and copy number and their dependence on one another are not well understood. Because of the tandem nature of the repeats in the rDNA array, the high rates of transcription it needs to accommodate, and the difficulty of replicating repetitive sequences, the rDNA array is highly prone to double stranded breaks (DSBs) and homologous recombination, which leads to loss of repeats at a rate as high as 1 copy per cell division [3,4]. In order to maintain rDNA copy number, the cell has at least two independent mechanisms to restore lost rDNA copies by amplification of repeats. These include unequal sister chromatid exchange, which occurs as a result of clearance of cohesin from rDNA by RNA polymerase II mediated transcription, and rolling circle replication [2,5,6].

Copy number of rDNA repeat units in eukaryotic cells is maintained far in excess of the requirement for ribosome biogenesis. In yeast, only about half of the ~150 rDNA repeats are actively transcribed to meet the translational needs of the cell [7]. Although only about half of these repeats are actively transcribed, it is known that in budding yeast, reduced copy number makes cells more sensitive to DNA damage [3].The extra, untranscribed copies are thought to contribute to stability and integrity of the locus by acting as a ‘foothold’ for repair enzymes and for contacts with other regions of the genome [3,8–10]. Human population genome sequencing data analysis has revealed correlations between rDNA copy number and the expression of several genes encoding chromatin-modifying proteins [11], further supporting the idea that the effects of rDNA copy number go well beyond rRNA production for ribosome biogenesis. These could involve, for example, differential recruitment of chromatin-modifying proteins to rDNA arrays with variable copy number. Disproportional binding of chromatin-modifying proteins to the rDNA locus could result in altered concentrations of these proteins throughout the rest of the genome, affecting chromatin environments and transcription genome-wide [12–14].

The hypervariablity of the rDNA locus combined with its ability to titrate various factors gives it the potential to act as a sensor of various conditions or stresses and in response, be a rapid and reversible source of variation for adaptation at the cellular and organismal levels. Several genetic factors have been identified that affect copy number. Of these, some, for example, Rtt109, a histone acetyltransferase, regulate rolling-circle replication of rDNA repeats [2], and others, like Rrn10, may act by regulating RNA polymerase I transcription [15]. On the other hand, some proteins like Sir2 and Fob1 are part of the pathway that senses loss of rDNA repeats and feeds back into regulation of recombination and replication, thereby ensuring that the array does not contract beyond a certain size [5,16]. Although the advantages of having additional copies of rDNA repeats, and the existence of mechanisms to regulate the amplification of rDNA repeats are becoming evident, very few studies have focused on the factors that determine “normal” copy number. Ide et al. [17] first reported that the rDNA array is hypersensitive to perturbations in initiation of DNA replication, and that rDNA array size could modulate cellular response to such perturbations. A more recent study by Kwan et al. [18] also showed that rARS activity affects rDNA copy number as well as genome-wide DNA replication dynamics. Although these studies support the idea that the rDNA array could act as a sensor and source of adaptive response to stress, identification of the mechanisms that determine normal rDNA array size requires an unbiased screen for factors that affect rDNA copy number.

To measure rDNA copy number in an accurate manner that is easily adaptable to high-throughput methods, we developed a droplet digital PCR (ddPCR) based assay to measure rDNA copy number in yeast and used it to identify essential genes involved in the maintenance of rDNA copy number. We screened the 787 mutants of the yeast conditional temperature-sensitive (ts) mutant collection of essential genes [19] for mutants with altered rDNA copy number. Our data revealed that low rDNA copy number is associated with compromised DNA replication. Consistent with this, we found that subculturing yeast under two separate conditions of DNA replication stress causes a contraction of the rDNA array independent of the replication fork blocking protein, Fob1. Additionally, we show that a smaller rDNA array enables cells to complete DNA replication in a timely manner, likely by freeing scarce replication factors for use by the rest of the genome, thereby allowing cells to survive and adapt to DNA replication stress. Finally, the loss of rDNA repeats in thymic tumors derived from mice with reduced expression of mini chromosome maintenance 2 (MCM2), a key component of the MCM2-7 DNA replicative helicase, also suggests that rDNA copy number may be used as an indicator of past stress, and that past replication stress may be diagnosed by a contracted rDNA array. Taken together, these data begin to suggest the selective pressures that combine to yield a “normal” rDNA copy number.

## Results

### Development and validation of a ddPCR assay to measure rDNA copy number in yeast

**Fig 1:**
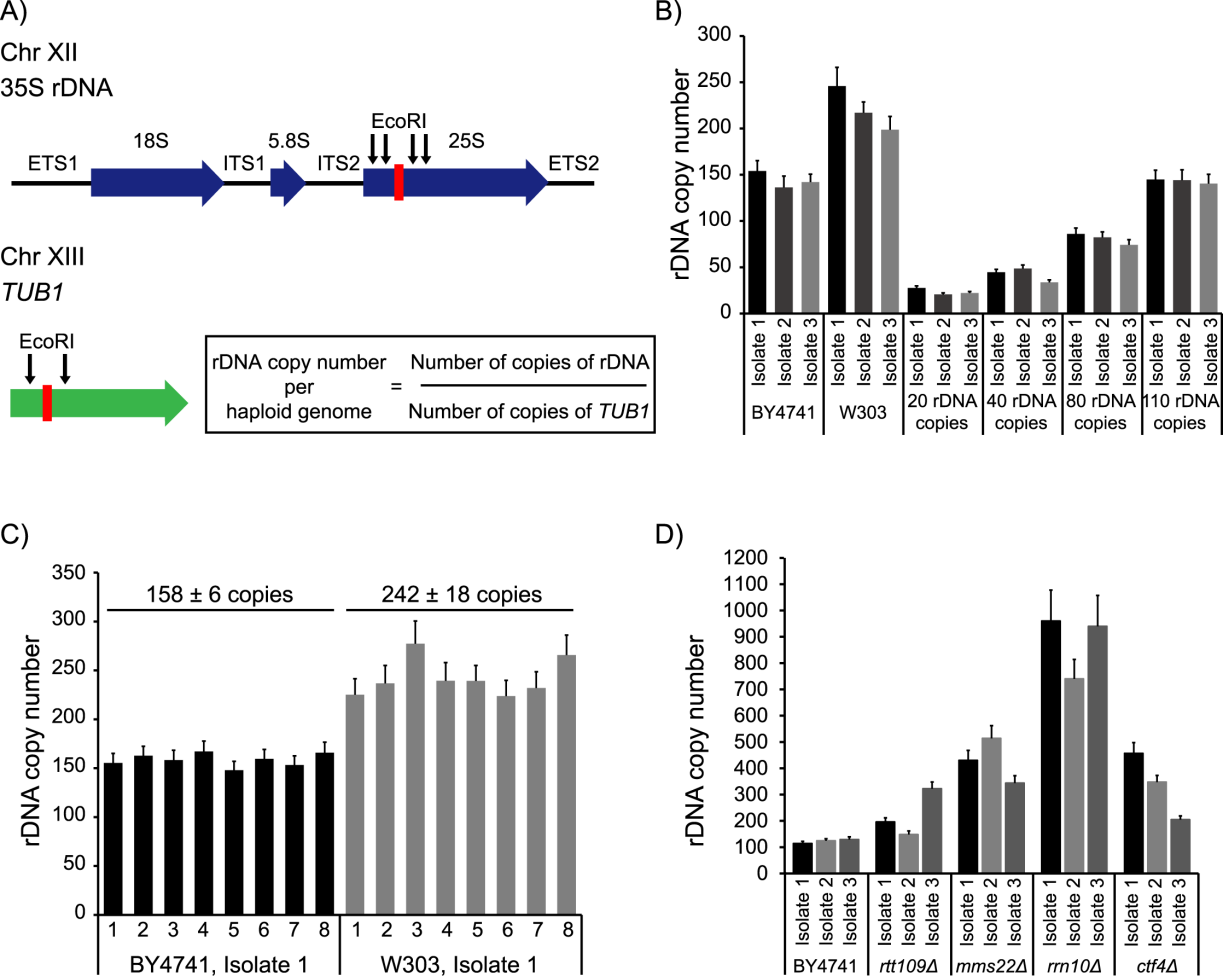
Design and validation of a ddPCR assay for rDNA copy number measurement. (A) Targets (red bars) within the rDNA and single copy reference (*TUB1*) loci in yeast. (B) rDNA copy number in 3 independent isolates each of 2 different wild-type laboratory yeast strains, BY4741 and W303, and 4 isogenic strains with varying rDNA copy number generated by Ide et al. [3]. (C) rDNA copy number in 8 technical replicates each of BY4741 and W303. (D) rDNA copy number in 3 independent isolates each of mutants with expanded rDNA arrays. Error bars represent standard deviation for each individual reaction.

Rapid and accurate high-throughput characterization of the genetic determinants of rDNA copy number has thus far been impeded by the lack of simple, sensitive assays to measure copy number. Previous methods of determining rDNA copy number involved measuring changes in the size of chromosome XII by Pulsed Field Gel Electrophoresis (PFGE) and subsequent Southern blotting [20]. More recently, qPCR has been used to measure copy number [21,22]. However, adapting qPCR to high-throughput experiments is difficult because of the requirement for a standard curve for each experiment, and its ability to detect only relatively large changes in copy number. ddPCR is a DNA molecule-counting technique that directly counts the absolute number of DNA molecules in a sample. In ddPCR, targets of interest are partitioned into ~20,000 nano-droplets and amplified to endpoint with TaqMan probes as in qPCR. The concentrations of the targets are then determined by counting the number of fluorescently positive and negative droplets in the sample. Thus, the fluorescence signal in a qPCR is converted from an analog signal into a digital one in ddPCR, eliminating the need for standard curves and allowing the determination of target copy number on an absolute scale with high precision [23]. In our assay, primers and fluorescent probes specific to the 25S coding sequence of the rDNA repeat, and to a stable, essential, single copy control gene, *TUB1* were designed (Fig 1A). Absolute copy numbers of each target in the sample are obtained by a duplexed ddPCR reaction, and rDNA copy number per haploid genome calculated from their ratio.

The assay was validated by measuring rDNA copy number in multiple independent isolates of two wild-type laboratory yeast strains, BY4741 and W303, and strains with 20–110 rDNA copies, in which rDNA copy number has been previously measured using PFGE [3]. BY4741 has ~150 rDNA copies, and W303 has ~250 rDNA copies, and the strains with 20–110 rDNA copies were also easily distinguishable (Fig 1B). To estimate the range of technical error, we measured rDNA copy number in 8 technical replicates each of both wild-type strains, BY4741 and W303. In our assay, technical error is within 10%, and often within 5% (Fig 1C). Since each reaction is partitioned into ~20,000 droplets, technical error in an individual reaction can be calculated based on the droplet data from that well. Technical error in ddPCR comes mainly from errors due to sub-sampling, and partitioning into droplets. In a good assay, this total technical error should be close to the standard error of the mean, and in our assay, closely matches the error from true technical replicates (Fig 1C). Therefore, we use the errors calculated for each individual reaction as an estimate of technical error, which eliminates the requirement for multiple technical replicates per sample. Biological replicates, however, are critical due to natural biological variability in rDNA copy number. Finally, we also used our ddPCR assay to confirm an increased rDNA copy number in 3 independent isolates each of *rtt109Δ*, *mms22Δ*, *rrn10Δ*, and *ctf4Δ* strains, which were previously reported to have expanded rDNA arrays [2] (Fig 1D).

### Identification of essential genes involved in maintaining rDNA copy number

**Fig 2:**
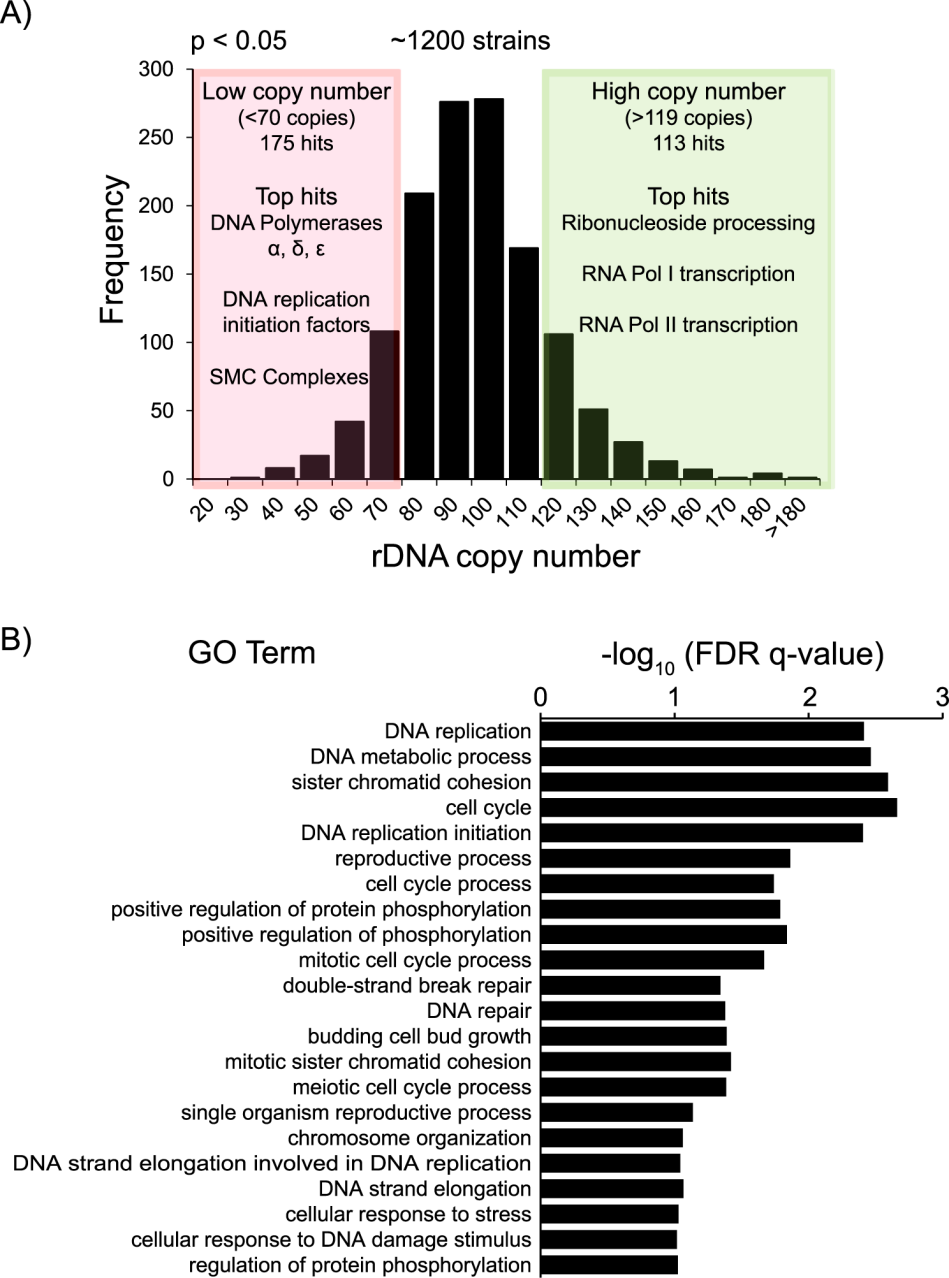
Screen for essential genes that maintain rDNA copy number. (A) Distribution of rDNA copy number across the yeast ts mutant collection. 175 and 113 strains had significantly lower (<70 copies) and higher copy number (>119 copies) respectively (p<0.05). (B) -log_10_ transformed FDR q-values for significantly enriched (p<0.001) GO terms (sorted in order of increasing p-values from top to bottom) in low copy number hits.

To screen for essential genes involved in the maintenance of rDNA copy number, we used the ddPCR assay to measure rDNA copy number in high-throughput format in the yeast ts mutant collection of essential genes [19]. This collection contains 787 ts strains, covering 497 (~45%) of the 1,101 essential yeast genes, with ~30% of the genes represented by multiple alleles. The mutants, along with wild-type controls, were grown at the permissive temperature (room temperature), and then shifted to the restrictive temperature (37°C) for 3 hours. Following this, genomic DNA was isolated for ddPCR. A distribution of rDNA copy number across the ~1200 strains screened is shown in Fig 2A. The mean rDNA copy number of wild-type strains was 95 ± 12.175. Mutants with significantly higher or lower rDNA copy number were identified based on thresholds set by variation in rDNA copy number in wild-type controls (Fig 2A). Of the ~1200 strains screened, 288 had significantly altered copy number (p < 0.05). The top 89 hits in each category, low and high copy number, were further validated by measuring rDNA copy number in two additional biological replicates (S1 Table). These top hits were also tested for aneuploidies in chromosomes XII and XIII, on which the ddPCR targets reside (S1 Table). Only 5 hits, YDR311W (*tfb1-1*), YGR002C (*swc4-4*), YIL126W (*sth1-2*), and YLR186W (*emg1-1*), and YNR053C (*nog2-1*) had a ratio of Chromosome XII to Chromosome XIII that may be representative of aneuploidies that could affect rDNA copy number measurements, and these mutants were excluded from all further analyses. Additionally, rDNA copy number was also measured at the permissive growth temperature to ensure that the restrictive temperature itself did not confound our results. In fact, we found that the hits with altered rDNA copy number after growth at the restrictive temperature also had similarly altered copy number even at the permissive temperature (S1 Table), suggesting that the effects on rDNA array size are likely a result of prolonged propagation in the mutant background.

Gene Ontology (GO) enrichment analyses revealed a striking difference in the pathways associated with low or high copy number. Most of the hits with high copy number were mutants in transcription and RNA processing genes, however none of these GO terms were significantly enriched in our hits relative to their background frequencies in the ts mutant collection. On the other hand, the hits with low rDNA copy number were significantly enriched for DNA replication mutants (p<0.001) (Fig 2B). Further examination of the “low copy number” hits revealed that the most significantly enriched hits were mutants in subunits of DNA polymerases α, δ, and ε, and various replication initiation complexes, such as the Mini chromosome maintenance 2–7 (Mcm2-7) complex, the Origin Recognition Complex (ORC), and the Cdc7-Dbf4 complex. The “low copy number” hits were also significantly enriched for mutants in subunits of the cohesin complex, well known for its multiple roles in maintaining chromosome integrity, including DNA replication and repair [24–26]. Therefore, this data suggested that compromised DNA replication may select for contraction of the rDNA array.

### DNA replication stresses cause contraction of the rDNA array independent of Fob1

**Fig 3:**
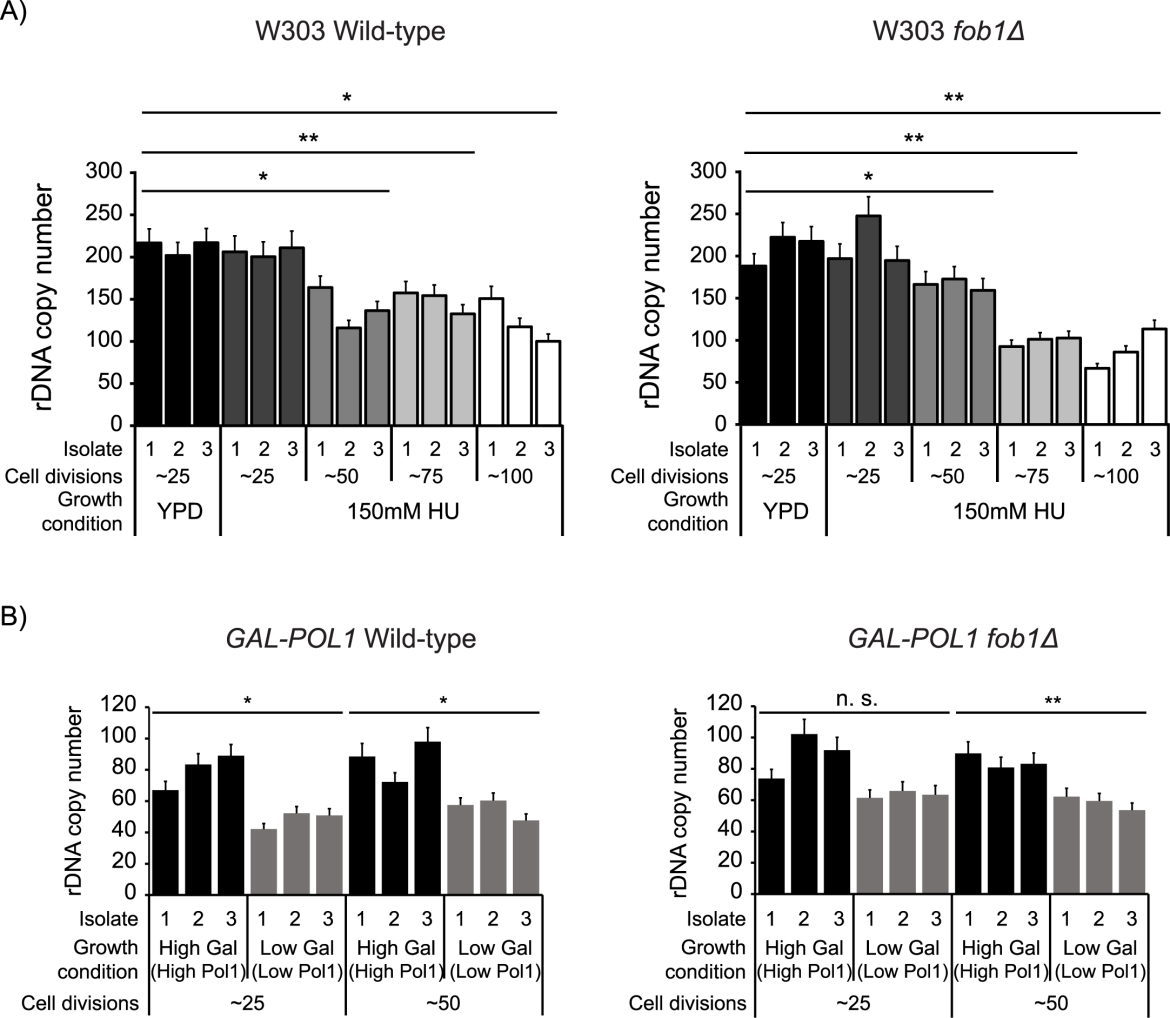
DNA replication stresses cause a contraction of the rDNA array independent of Fob1. rDNA copy number in A) WT or *fob1Δ* W303 cells subcultured in 150mM hydroxyurea (HU) for ~100 generations, and B) WT or *fob1Δ* strains with *POL1* driven by galactose inducible *GAL1-10* promoter subcultured in medium containing high or low galactose for ~50 generations. After every 25 generations, 3 independent isolates were used to measure rDNA copy number by ddPCR. Error bars represent standard deviation for each individual reaction. Statistical significance was calculated using a standard 2 tailed t-test. *—p<0.05, **—p<0.01, n. s.–non-significant.

Recently Kwan et al. [27] reported that unsolicited, and stably maintained rDNA copy number variations resulting from exposure to lithium acetate were prevalent in yeast mutant collections generated by standard transformation protocols. Although it is still unclear whether exposure to lithium acetate induces unsolicited rDNA copy number variations or simply selects for pre-existing copy number variations, it is possible that the copy number changes in at least a subset of our hits are a result of transformation protocols, and unrelated to the gene mutation. As a preliminary validation of the correlation between the gene mutation and the change in rDNA copy number in our hits, we analyzed the copy number changes in mutant strains of genes with multiple ts alleles, or multiple isolates of the same ts allele. We found that for most genes with validated rDNA copy number changes, a majority of the multiple mutant strains had a similar alteration in rDNA copy number (S1 Table). This is a strong indication that at least for a subset of genes, the mutation itself is likely responsible for the altered rDNA array size. To further validate the findings from our screen, we subcultured yeast cells under two separate conditions of DNA replication stress by a) plating them on medium containing 150mM hydroxyurea (HU), a ribonucleotide reductase inhibitor, or b) depleting Pol1, the largest subunit of DNA polymerase α, using a diploid strain homozygous for the *GAL-POL1* allele, containing galactose inducible Pol1 [28]. Both of these conditions caused a contraction of the rDNA array after 50–75 generations, independent of Fob1 (Fig 3). Additionally, *rnr1Δ* mutants lacking Rnr1, the major isoform of the large subunit of ribonucleotide-diphosphate reductase also have contracted rDNA arrays (S1 Fig). These findings led us to hypothesize that since the rDNA is one of the most unstable and hypervariable loci, and difficult to replicate, DNA replication stresses select for smaller rDNA arrays, perhaps because cells with lower copy number can complete DNA replication in a timely manner and continue to propagate. This raised two main questions– 1) What are the consequences of having a contracted array? 2) Does a smaller rDNA array help cells survive and adapt to DNA replication stress, and if so, how?

**Fig 4:**
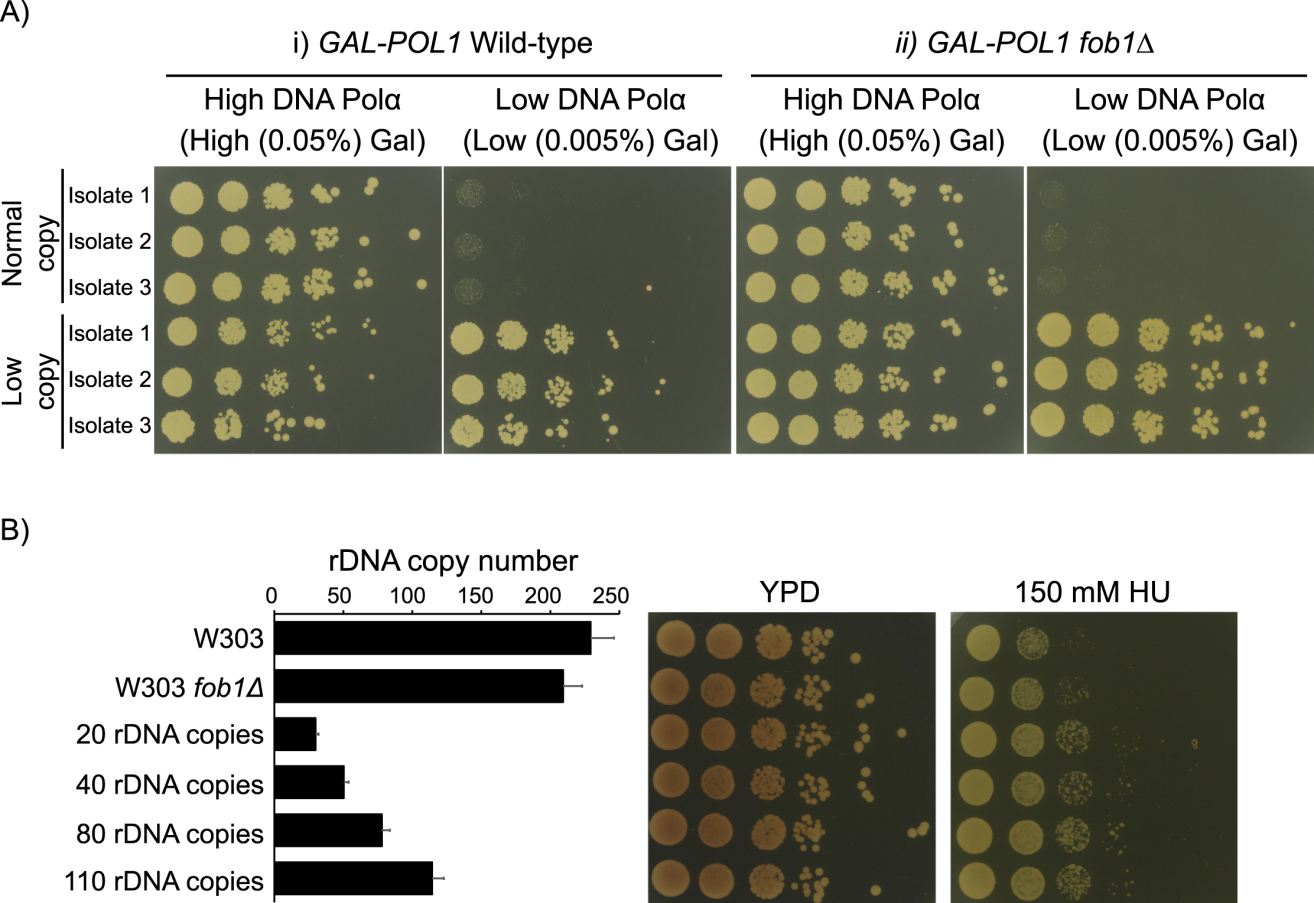
Low rDNA copy number confers advantage under DNA replication stress. (A) 3 independent isolates each with normal or low rDNA copy number were generated by subcultuing wild-type or *fob1Δ GAL-POL1* cells in medium containing high or low galactose (high or low levels of Pol1 respectively) for ~50–75 generations (S2 Table) and 5-fold serial dilutions spotted on to medium with DNA replication stress (low Pol1). (B) 10-fold serial dilutions of wild-type and *fob1Δ* cells (~200–250 rDNA copies) along with cells having 20–110 rDNA copies [3] were spotted on medium containing hydroxyurea (HU). rDNA copy number was confirmed by ddPCR. Error bars represent standard deviation for each individual reaction.

To address these questions, we used the strain with galactose inducible Pol1 to generate cells with normal or low rDNA copy number as in Fig 3B by growing the strain in medium containing either high or low galactose (high or low DNA polymerase α) respectively. Given the isolate to isolate variability in rDNA copy number, we generated and used 3 independent isolates from each condition for all our studies. Ide et al. [3] reported that low rDNA copy number makes cells more sensitive to DNA damaging agents, such as UV and methyl methanesulfonate (MMS). We found that wild-type, but not *fob1Δ* isolates with low rDNA copy number generated by Pol1 depletion also exhibited mild sensitivity to bleomycin and UV (S2 Fig). Notably, both wild-type and *fob1Δ* isolates with low rDNA copy number grew better than those with normal copy number under conditions of DNA polymerase α depletion (Fig 4A). However, selection under altered levels of DNA polymerases is known to induce general genome instability, and often results in mutations that restore normal expression levels [29,30]. Therefore, we wanted to ensure that the survival of the low copy number isolates generated by selection under low levels of DNA polymerase α was not due to chromosomal abnormalities or other mutations which enable expression of normal levels of Pol1. Song et al. [29] showed that trisomy of Chromosome XIV (on which *POL1* resides) is very rare after 50–75 generations of selection under low levels of DNA polymerase α. Additionally, we tested for deletions in the *GAL1-10* promoter in the normal and low copy number isolates by a PCR-based strategy [30], and found that the *GAL1-10* promoter was intact after 50–75 generations of selection (S3 Fig). Additionally, strains with low rDNA copy number (20–110 rDNA copies, [3]) are also less sensitive to HU than corresponding wild-type cells (~200–250 rDNA copies) (Fig 4B) supporting the hypothesis that low rDNA copy number is advantageous to cells under conditions of DNA replication stress.

### Contraction of the rDNA array enables timely completion of DNA replication and cell cycle progression

**Fig 5:**
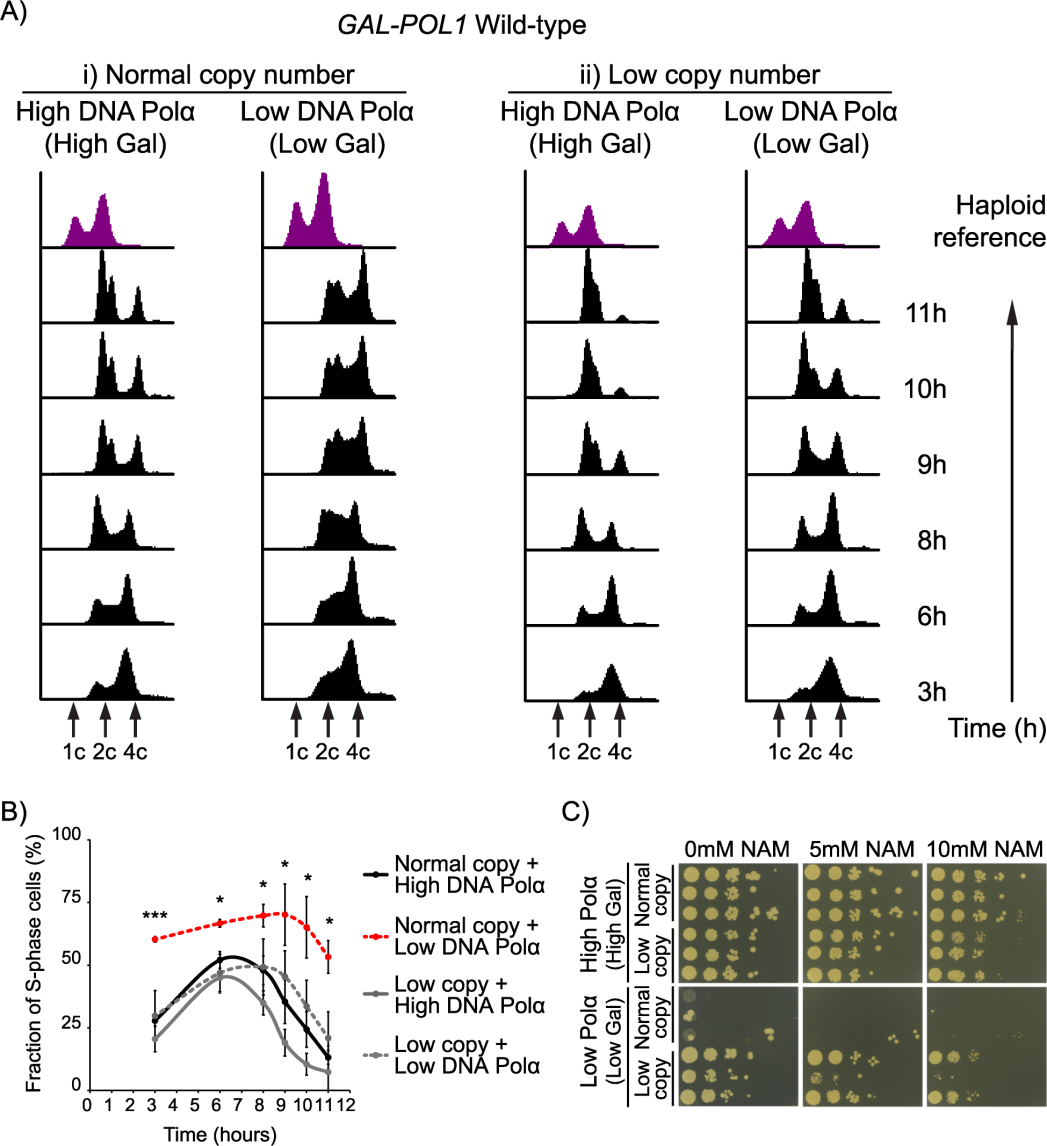
Contraction of the rDNA array promotes timely completion of DNA replication and cell cycle progression. Wild-type *GAL-POL1* cells were subcultured in medium containing high or low levels of galactose for ~75 generations to generate 3 independent isolates each with normal or low rDNA copy number (S2 Table). (A) Representative DNA content profiles over time are shown for asynchronous cultures of isolates with normal (i) and low (ii) rDNA copy number following inoculation into the indicated medium which determines high or low levels of DNA polymerase α. (B) Fraction of cells in S-phase in each of the 4 conditions in (A). Error bars indicate standard deviation based on 3 independent isolates. Statistical significance of differences between fraction of cells in S-phase in high and low levels of DNA polymerase α was calculated using a standard 2-tailed t-test. *—p<0.05, ****—p<0.0001. (C) Increased rARS firing in nicotinamide exacerbates growth defects under conditions of DNA replication stress.

To further characterize how low rDNA copy number helps cells survive DNA replication stress, we measured the fraction of cells in S-phase in asynchronous cultures of both low and normal copy number isolates under conditions of both high and low levels of DNA polymerase α by flow cytometry. We found that with low levels of DNA polymerase α, the high copy number isolates show a higher fraction of cells in S-phase compared to their low copy number counterparts (Fig 5A and 5B, S4A and S4B Fig). This shows that under conditions of DNA replication stress, cells with smaller rDNA arrays are able to proceed through S-phase and complete DNA replication in a timely manner, allowing them to continue to propagate.

To test the role of the rDNA array in this rescue, we grew these isolates in high/low galactose (high/low levels of DNA polymerase α respectively) medium containing nicotinamide (NAM), a Sir2 inhibitor. Sir2 represses rARS firing and Sir2 inhibition by NAM will cause more rARS firing [31]. One possible effect of increased rARS firing is the titration of replication factors away from the rest of the genome, which could make the normal copy number strain grow poorly, even with high levels of DNA polymerase α. Another consequence of increased rARS firing in NAM is that the distance each replication fork has to travel in the rDNA will be shorter, which might instead rescue the normal copy number strain with low levels of DNA polymerase α. We found that NAM caused the isolates with normal rDNA copy number to grow poorly with low levels of DNA polymerase α (Fig 5C, S4C Fig). We also observed that the isolates with low rDNA copy number with low levels of DNA polymerase α exhibited mild sensitivity to higher concentrations of NAM (Fig 5C, S4C Fig). Since higher concentrations of NAM could also inhibit other sirtuins, including Hst3 and Hst4, which are known to participate in DNA replication [32], it is possible that the phenotypes observed in NAM are due to a combination of increased rARS firing and additional DNA replication stress imposed by the inhibition of Hst3 and Hst4. Altogether, this suggests that titration of already scarce replication factors by the rDNA is the primary issue faced by the normal copy isolates under conditions of DNA replication stress.

### DNA replication stress may be diagnosed by rDNA array size

**Fig 6:**
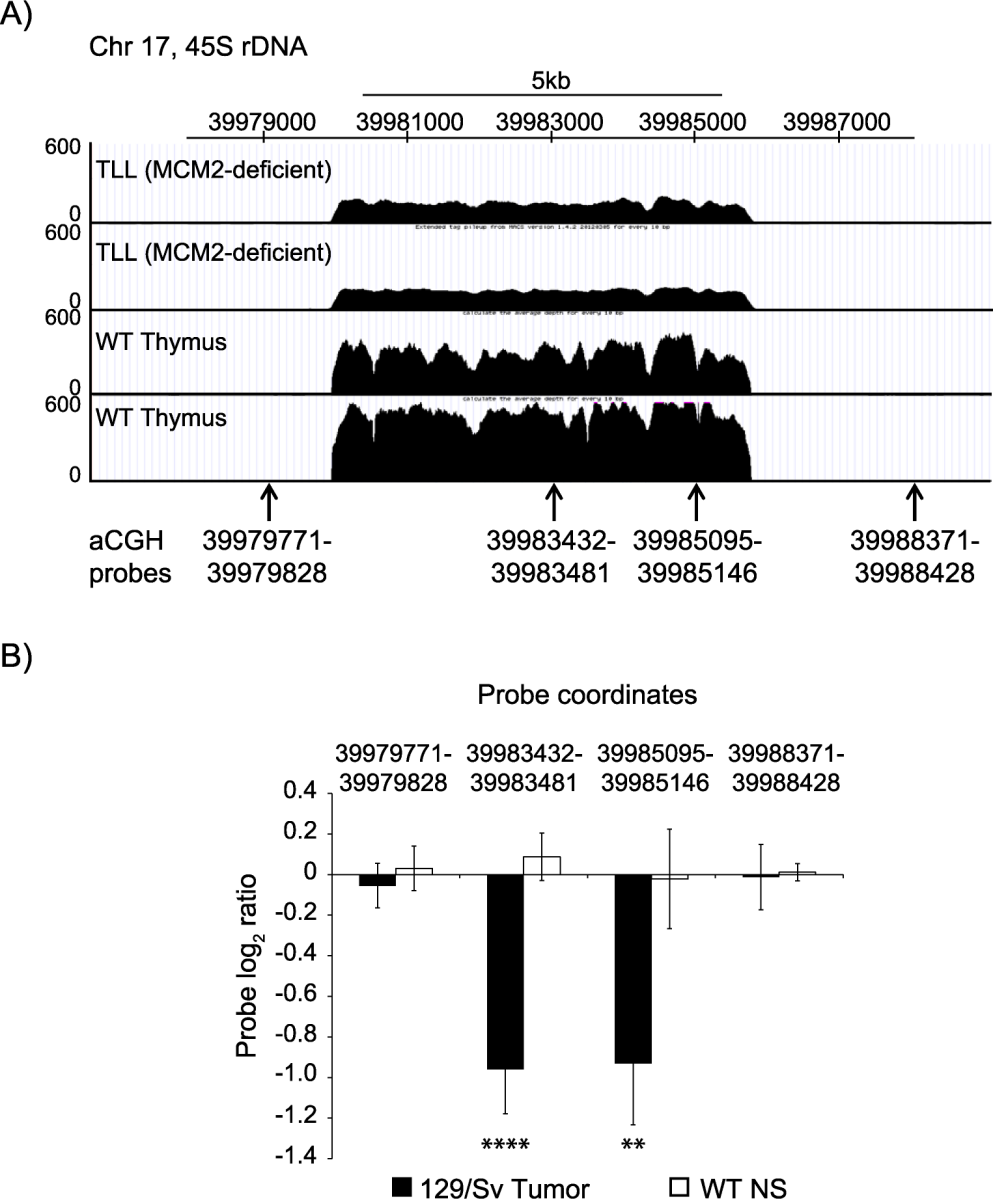
45S rDNA repeats are lost in T-lymphocytic leukemia (TLL) tumors of MCM2-deficient mice. (A) Sequence tag densities (normalized for number of mapped reads) over a portion of the 45S rDNA from 2 TLL tumors arising in *Mcm2*^*IRES-CreERT2/IRES-CreERT2*^ mice on a 129/Sv x C57Bl/6 F1 genetic background and 2 thymuses from wild-type littermates [39]. (B) Average aCGH log_2_ values for probes from (A) for 8 TLL tumors arising in *Mcm2*^*IRES-CreERT2/IRES-CreERT2*^ 129/Sv mice [38] compared to average aCGH log_2_ values for the same probes from 3 clonally derived neural stem cell cultures from wild-type littermates (WT NS). Error bars indicate standard deviation. Statistical significance (129/Sv tumor vs. WT NS) was calculated using a standard 2 tailed t-test. **—p<0.01, ****—p<0.0001.

Our data show that in yeast, under conditions of DNA replication stresses, a smaller rDNA array provides a selective advantage to cells. Since persistent DNA replication stress resulting from mutations that affect DNA replication machinery have been associated with both cancer [33] and human developmental syndromes [34], we wondered whether these human conditions would also be associated with genomes containing contracted rDNA arrays. To explore this, we used a previously established MCM2-deficient mouse model of cancer, generated by integration of a tamoxifen-inducible form of Cre recombinase downstream of the *Mcm2* coding sequence and expression via an internal ribosome entry site (IRES) [35]. In this model, homozygous *Mcm2*^*IRES-CreERT2/IRES-CreERT2*^ (MCM2-deficient) embryos or mouse embryonic fibroblasts have reduced MCM2 expression, approximately one-third of wild-type levels. Although these mice develop normally, MCM2-deficiency results in stem cell deficiencies in multiple tissues, and ultimately, cancer, primarily lymphomas [35]. Moreover, it was also recently reported that the 45S rDNA repeats accumulate high levels of DNA damage in the form of DSBs, both in these MCM2-deficient mice [36], as well as in mouse hematopoietic stem cells (HSCs) with reduced MCM expression [37]. Therefore, we used array-based Comparative Genome Hybridization (aCGH) and Next Generation Sequencing (NGS) to study rDNA copy number in thymic lymphoblastic lymphoma (TLL) tumors derived from MCM2-deficient mice. We performed aCGH on 8 TLL tumors derived from *Mcm2*^*IRES-CreERT2/IRES-CreERT2*^ mice on the 129/Sv (6 of 8 tumors) or 129/Sv x C57Bl/6 F1 (2 of 8 tumors) genetic backgrounds using the NimbleGen 720K whole-genome tiling arrays [38]. All samples were assessed relative to DNA from non-tumorous tissue derived from wild-type littermates of the same genetic background. We also performed NGS on 2 TLL tumor samples derived from *Mcm2*^*IRES-CreERT2/IRES-CreERT2*^ mice on a 129/Sv x C57Bl/6 F1 genetic background and control thymic DNA samples from 2 wild-type littermates. Comparison of aCGH probe log_2_ ratios for TLL tumor DNA and DNA from 3 clonally derived neural stem cell cultures along with the NGS data confirmed a loss of approximately half of the 45S rDNA repeats in MCM2-deficient tumors (Fig 6). Although the loss of rDNA repeats may simply be a byproduct of tumorigenesis, and not a direct consequence of MCM2 deficiency, these data suggest that under conditions of genotoxic stress, the rDNA may be highly susceptible to DSBs, and while both gain and loss of rDNA repeats may occur frequently in the face of the DNA damage, losses may be selected for under conditions of DNA replication stress. This suggests that rDNA array size may be used as a molecular diagnostic marker for past DNA replication stress.

## Discussion

The rDNA array is the most unstable, and hypervariable genetic locus in the yeast genome. It is the most highly transcribed genomic locus, difficult to replicate owing to its repetitive nature, and comprises over 60% of chromosome XII, and ~10% of the yeast genome. This unique locus, therefore, has the potential to act as the “canary in the coalmine”, being particularly sensitive to stresses that disrupt genome integrity, and acting as a source of cellular adaptation, relatively simply, through variation in array size. In wild-type yeast cells, rDNA repeat copy number is maintained at ~150 per haploid cell. However, owing to the tandem arrangement of the repeats, and the associated instability, repeats are lost at a relatively high rate of up to ~1 per cell division [4]. Therefore, there must be mechanisms in place to a) sense rDNA copy number at each cell division, and b) trigger an appropriate response so that the array size can be maintained at wild-type levels. Studies so far have revealed many pathways that contribute to the maintenance of the rDNA locus, but the fundamental question of what determines normal repeat copy number remains unanswered.

Our screen has identified essential proteins involved in maintenance of the rDNA locus based on screening 45% of essential yeast genes; in the future, we can extend this to nearly 75% by screening additional mutant collections [40]. Our data show that DNA replication stresses select for cells that have a smaller rDNA array, and a smaller array helps cells adapt to replication stress. Data from several studies support our findings–i) contraction of the rDNA array can rescue yeast temperature-sensitive mutants of the Origin Recognition Complex, the key replication initiation complex [17], ii) a large deletion of the rDNA array can rescue the synthetic growth defects of *rif1Δ* cells lacking either MRX or Ctf4-Mms22 activity [41], iii) a polymorphism in the rARS that results in a weakly replicating rDNA array causes a contraction of the array and promotes DNA replication in the rest of the genome [18], and iv) coordination of DNA replication and recombination at the rDNA is critical to maintain the integrity and size of the rDNA array [42]. Our data supports the previously reported role of extra, untranscribed rDNA repeats in protecting cells from DNA damaging agents [3]. In addition, our data suggests that while extra, untranscribed rDNA repeats are essential for allowing co-transcriptional DNA damage repair, they become detrimental to cells under conditions of DNA replication stress.

Each rDNA repeat unit consists of a DNA replication origin, the rARS, and the rDNA array represents roughly one-third of all replication origins in the yeast genome [43]. At any given time in S-phase, only a fraction of licensed origins are fired, and this is thought to be achieved by limiting the pool of initiation factors available [44]. Recently, Foss et al. [43] presented evidence to support the long standing idea that, in yeast, repetitive rDNA compete for origin firing with unique genomic sequences (in yeast, the rest of the genome), and tipping the balance in favor of repetitive rDNA can lead to replication gaps, or underreplicated regions, throughout the rest of the genome. Our data also show that under conditions of DNA replication stress, cells with a smaller rDNA array are able to complete DNA replication and proceed through the cell cycle. This, combined with the growth defects of the cells with normal rDNA copy number in NAM, is in agreement with Foss et al.’s observation that replication gaps produced by deletion of *SIR2* can be suppressed by decreased origin activity within the rDNA. Taken together, these data suggest that the repetitive rDNA array may be particularly sensitive to DNA replication stresses, and while DNA damage repair could result in a gain or loss of repeats, the loss of repeats provides a selective advantage by liberating scarce DNA replication factors for use by the rest of the genome.

Finally, we speculate that the size of the rDNA array may be used as an indicator of past stress. This has interesting implications for human disease. The link between persistent DNA replication stress and tumorigenesis is well established [33]. Additionally, mutations in key components involved in initiation of DNA replication, such as ORC1, ORC4, ORC6, CDT1, CDT6, and CDC45, have been reported to be the cause of Meier-Gorlin syndrome, a primordial dwarfism syndrome [34,45]. Our data suggest that these human conditions will be associated with genomes containing contracted 45S rDNA arrays. Xu et al.[46] and Wang and Lemos [47] recently showed through bioinformatic analyses of whole genome sequencing data from various cancers that 45S arrays are often lost in cancer. These cancer genomes show evidence of a hyperactive mechanistic target of rapamycin (mTOR) pathway [46] and p53 mutations [47]. Xu et al. further discovered that mouse HSCs lacking PTEN, a negative regulator of mTOR, also had contracted 45S rDNA arrays, and these cells were more sensitive to DNA damaging agents such as bleomycin, MMS, and X-rays. Interestingly, this DNA damage sensitivity was independent of mTOR activity, and mainly attributed to low rDNA copy number. Although PTEN is a phosphatase widely known for its role as a tumor suppressor, nuclear PTEN is essential for maintaining genome stability by dephosphorylating MCM2, and modulating replication fork progression under conditions of DNA replication stress [48], suggesting that the loss of 45S rDNA repeats observed in the *Pten*^*-/-*^ mouse HSCs could be attributed to DNA replication stress. DNA replication stress resulting from reduced MCM expression was also shown to drive functional decline in aging mouse HSCs, and caused accumulation of phosphorylated γH2AX at the rDNA [37]. Our findings on the effect of DNA replication stress on the yeast rDNA predict that persistent DNA replication stress would select for a contraction of the rDNA array in each of these cases, and this is in fact what we observe in the tumors from MCM2-deficient mice. Consistent with previous reports, mouse embryonic fibroblasts derived from our MCM2-deficient mice also show increased levels of DNA damage at the 45S rDNA repeats and sensitivity to UV [49]. This is compelling evidence to suggest that DNA replication stress may be diagnosed by rDNA copy number, and cells with low copy number may be more susceptible to common DNA damaging chemotherapeutic agents. Therefore, rDNA copy number may prove to be an important indicator in human disease. Altogether, these results provide insight into the role of the rDNA locus in acting as a sensor and source of rapid, reversible adaption to general genomic stress through a plastic array size determined by selective cues from the environment.

## Materials and methods

### Yeast strains and growth media

All yeast strains used are listed in S3 Table. Unless otherwise stated, all growth was carried out in YPD (1% w/v yeast extract, 2% w/v peptone, 2% w/v dextrose) at 30°C. For the experiment in Fig 3A, cells were grown in YPD containing 150mM hydroxyurea. The *GAL-POL1* strains were grown in YPR (1% w/v yeast extract, 2% w/v peptone, 3% w/v raffinose) medium containing either high (0.05% w/v) or low (0.005% w/v) levels of galactose as indicated.

### rDNA copy number measurement by ddPCR

Genomic DNA was isolated using the YeaStar Genomic DNA Kit (Zymo Research). DNA concentrations were measured on a Qubit Fluorometer using the Qubit dsDNA HS Assay (Invitrogen). For ddPCR, 0.005ng genomic DNA was used per 20μL reaction. Primers and probes used are listed in S4 Table. Duplexed ddPCR was performed according to the manufacturer’s protocol (Bio-Rad). Briefly, master mixes containing primers and probes for rDNA and *TUB1*, genomic DNA, and the restriction endonuclease EcoRI-HF (New England Biolabs, Inc.) were prepared and aliquoted into Eppendorf twin.tec plates. Reaction mixtures were incubated at room temperature for 15 minutes to allow restriction digestion of genomic DNA prior to droplet generation. Droplets were cycled to endpoint and subsequently read using the QX200 droplet reader. Quantification was performed using the Quantasoft software. Standard deviation (SD) for each individual reaction was calculated using the formula

$$\mathrm{Standard}\;\mathrm{deviation}=\left(CI_{\max}\;\text{-}\;CI_{\min}\right)/\left(2\times 1.96\right)$$ (1)

where, (CImax—CImin) is the 95% Confidence Interval for the ratio of absolute copy number of rDNA and *TUB1* in each reaction, with both assays multiplexed in the same well, as generated by Quantasoft.

### High-throughput screen for mutants with altered rDNA copy number

#### 96-well plate re-array

The *Saccharomyces cerevisiae* ts mutant collection was re-arrayed from its source 384-well format into a 96-well format. This served the purpose of eliminating empty wells, which provided space for control strains (wild-type BY4741 and W303 (2 positions each, 1 fixed position across all plates, and 1 variable position), and the rDNA copy number control strains with 40, 80, and 110 rDNA copies [3] (one position each)), and eliminated the need for genomic DNA isolation from numerous empty wells present in the source 384-well format. The re-array was performed using the eight-channel independent liquid handling arm (LiHa) of a Tecan Freedom EVO liquid handling instrument by inoculating 10μL of the yeast ts glycerol stock into 150μL YPD + G418 (200mg/L) in a 96-well microtiter plate (Corning). These plates were incubated for 48 hours at room temperature, and then converted to glycerol stocks by adding 65μL of a 50% v/v mix of glycerol and YPD followed by mixing and freezing down at -80°C.

#### Genomic DNA isolation

Each re-arrayed 96-well plate was thawed and 30μL of each glycerol stock was inoculated into 1.6mL YPD + G418 (200mg/L) in a 96-deepwell plate (Thermo Fisher Scientific) and incubated at room temperature for 48 hours while shaking at 225rpm. The cultures were then incubated at 37°C for 3 hours while shaking at 225rpm. The plates were then spun down and medium was decanted. The cells were then re-suspended in 22μL of 3mg/mL Zymolyase 100T (US Biological) solution and incubated for an additional hour at 37°C while shaking at 225rpm. The plates were then spun down and the supernatant was decanted. Cell pellets were then processed to isolate genomic DNA using the ReliaPrep 96 genomic DNA MiniPrep HT System (Promega) on the Tecan Freedom EVO liquid handling instrument. The concentrations of genomic DNA in 96-well plates were measured using the QuantiFluor ONE dsDNA System (Promega) and read on a SpectraMax M2e plate reader (Molecular Devices). The genomic DNA was then diluted to 0.5ng/μL using the eight-channel independent LiHa of a Tecan Freedom EVO liquid handling instrument. The genomic DNA was subsequently diluted to 0.005ng/μL for ddPCR.

#### rDNA copy number measurement and hit determination

rDNA copy number across the ts mutant collection was measured using ddPCR as described above. In addition to the control strains on the ts mutant collection plates, an additional 96-well plate containing only the control strains (16 independent isolates per strain in randomized positions across the plate), BY4741, W303, the rDNA copy number control strains with 40, 80, and 110 rDNA copies, and YLR378C (*sec61-2*, the hit with the highest copy number across the ts mutant collection as measured in the initial screen) was also prepared for ddPCR to gauge variability in rDNA copy number between independent isolates, and any effects due to plate position. rDNA copy number measurements for BY4741 were used to set thresholds as follows: Mean rDNA copy number ± 1SD–No change. Mean rDNA copy number + 2SD > rDNA copy number > Mean rDNA copy number + 1SD OR Mean rDNA copy number - 2SD < rDNA copy number < Mean rDNA copy number - 1SD–Moderate change. rDNA copy number > Mean rDNA copy number + 2SD OR rDNA copy number < Mean rDNA copy number - 2SD–Significant change.

#### GO analyses

GO enrichment analyses were performed using Gorilla [50,51].

### Karyotyping

The top 89 hits of each category, low and high copy number, were cherry-picked, and genomic DNA was isolated from them in high-throughput format as described above. The genomic DNA was diluted to 0.5ng/μL and 0.01ng used per 20μL reaction for karyotyping. Partial karyotyping to obtain relative number of copies of Chromosomes XII and XIII was done using ddPCR. ddPCR assays for Chromosome XII and XIII were designed and duplexed to determine absolute copy number of each, which was subsequently used to calculate the ratio of chromosome XII relative to chromosome XIII. Primers and probes used, and their relative positions on chromosome arms are listed in S4 Table.

### Subculturing experiments

Strains were struck out on to appropriate medium from glycerol stocks. Single colonies were picked and re-streaked on to fresh plates every 2–3 days (approximately 25 cell divisions). At each subculture, additional single colonies were also used to isolate genomic DNA for ddPCR. All growth was at 30°C.

### Growth assays

Cells were diluted to a starting OD_600_ of 0.1 and 5 more 5-fold serial dilutions (unless otherwise mentioned), following which 4μL of each dilution was spotted on to plates containing the appropriate medium. Plates were incubated at 30°C for 2–3 days and photographed.

### Cell cycle analyses

3 isolates each with normal or low rDNA copy number were generated by growing wild-type or *fob1Δ GAL-POL1* strains on YPR medium containing either high or low levels of galactose for 50–75 generations. Following confirmation of rDNA array contraction by ddPCR (S2 Table), the isolates were each inoculated into liquid YPR medium containing either high or low levels of galactose. 100μL culture was collected at various time points. Cells were fixed using 70% v/v ethanol and treated with RNaseA, following which their DNA was stained using Sytox Green (1μM, at room temperature, in the dark, for at least 30 minutes). In parallel, wild-type haploid yeast cells for use as ploidy reference were also grown, fixed, treated with RNaseA, and stained with CellTrace Violet (1μM, at 37°C, in the dark, for 20 minutes, followed by 2 washes with 1x PBS) prior to staining with Sytox Green to allow distinction between reference and test strains. Before cytometric analysis, 20μL of the fixed and stained reference sample was added to 1mL of each fixed and stained test sample. DNA content analysis was performed on an EC800 Analyzer (Sony Biotechnology). DNA content modeling was performed using FCS Express 6 Plus (De Novo Software).

### aCGH and NGS

aCGH, NGS and subsequent data analysis were performed as previously described [38,39].

## Supporting information

S1 Fig rDNA copy number in *rnr1Δ* strains. rDNA copy number in 3 independent isolates each of BY4741 and *rnr1Δ* strains. Error bars represent standard deviation for each individual reaction. Statistical significance was calculated using a standard 2 tailed t-test. ***—p<0.001.
(TIF)

S2 Fig Low rDNA copy number makes cells sensitive to DNA damage. A) Wild-type or B) *fob1Δ GAL-POL1* cells were subcultured in medium containing high or low levels of galactose for ~50 generations to generate 3 independent isolates each with normal or low rDNA copy number (S2 Table). 5-fold serial dilutions of these isolates were spotted on to high galactose medium containing bleomycin, or spotted on to high galactose medium followed by irradiation with UV.
(TIF)

S3 Fig *GAL1-10* promoter is intact after 50–75 generations of selection in altered levels of Pol1. A) PCR scheme and relative positions of primers used [29]. B) Wild-type or *fob1Δ GAL-POL1* cells were subcultured in either high/low galactose. After ~50 generations, 3 independent isolates from each condition were used to isolate genomic DNA for PCR. PCR products were run on a 1% agarose gel. M– 100bp marker. *—Non-specific band.
(TIF)

S4 Fig Contraction of the rDNA array promotes timely completion of DNA replication and cell cycle progression in *fob1Δ*. *fob1Δ GAL-POL1* cells were subcultured in medium containing high or low levels of galactose for ~50 generations to generate 3 independent isolates each with normal or low rDNA copy number (S2 Table). (A) Representative DNA content profiles over time of asynchronous cultures of *fob1Δ* isolates with normal (i) and low (ii) rDNA copy number following inoculation into the indicated medium which determines high or low levels of DNA polymerase α. (B) Fraction of cells in S-phase in each of the 4 conditions in (A). Error bars indicate standard deviation based on 3 independent isolates. Statistical significance of differences between fraction of cells in S-phase in high and low levels of DNA polymerase α was calculated using a standard 2-tailed t-test. *—p<0.05, **—p<0.01. (C) Increased rARS firing in nicotinamide exacerbates growth defects under conditions of DNA replication stress.
(TIF)

S1 Table rDNA copy number, and karyotyping of Chromosomes XII and XIII in the top hits. The tabs labeled “Low copy number hits” and “High copy number hits” contain rDNA copy number measurements in 2 additional biological replicates, and at permissive temperature, and karyotyping of Chromosomes XII and XIII in the top low and high copy number hits respectively. The tabs labelled “Low copy multiple” and “High copy multiple” summarize rDNA copy number changes in mutants with multiple alleles/isolates in the low copy number and high copy number hits respectively.
(XLSX)

S2 Table rDNA copy number in *GAL-POL1* isolates used in Figs 4 and 5 and S2 and S4 Figs. (XLSX)

S3 Table List of yeast strains. (XLSX)

S4 Table Sequences of primers used. (XLSX)
