# Supplementary figures and images for: DNA replication stress restricts ribosomal DNA copy number
Source: PLoS Genet. 2017 Sep 15;13(9):e1007006. doi: 10.1371/journal.pgen.1007006 (PMC5617229; doi:10.1371/journal.pgen.1007006)

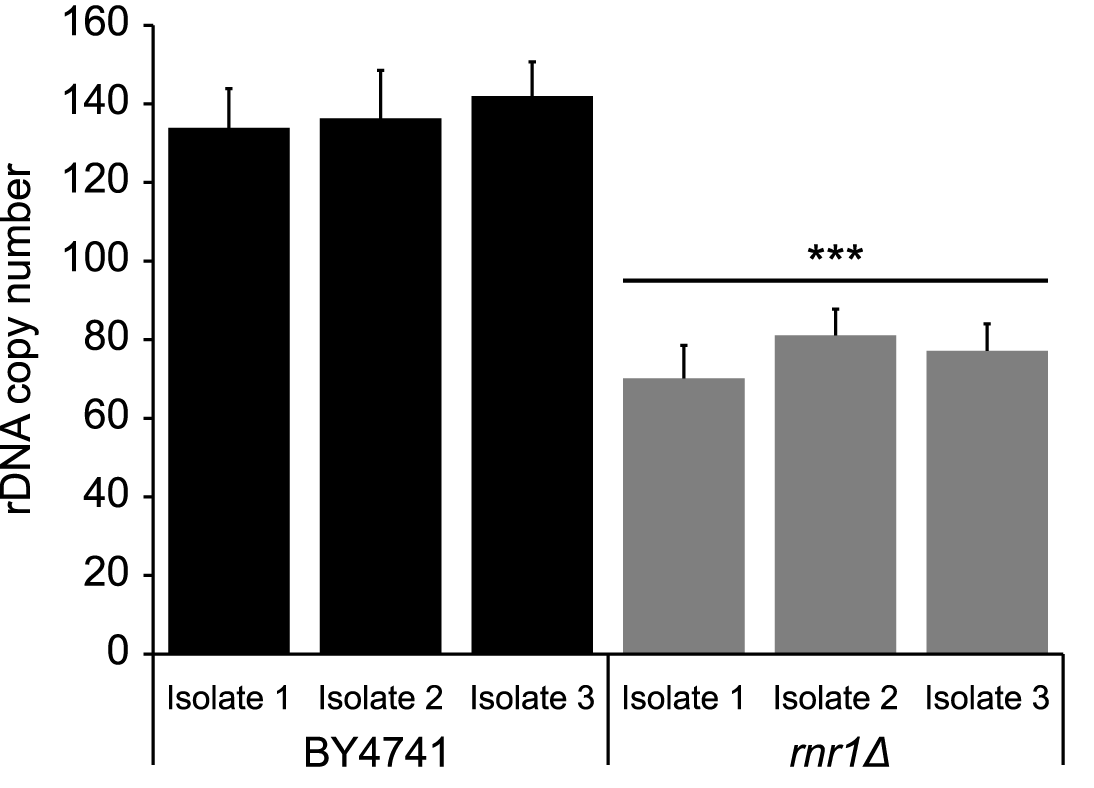

Supplement: S1 Fig — rDNA copy number in 3 independent isolates each of BY4741 and rnr1Δ strains. Error bars represent standard deviation for each individual reaction. Statistical significance was calculated using a standard 2 tailed t-test. ***—p<0.001. (TIF) [file pgen.1007006.s001.tif]

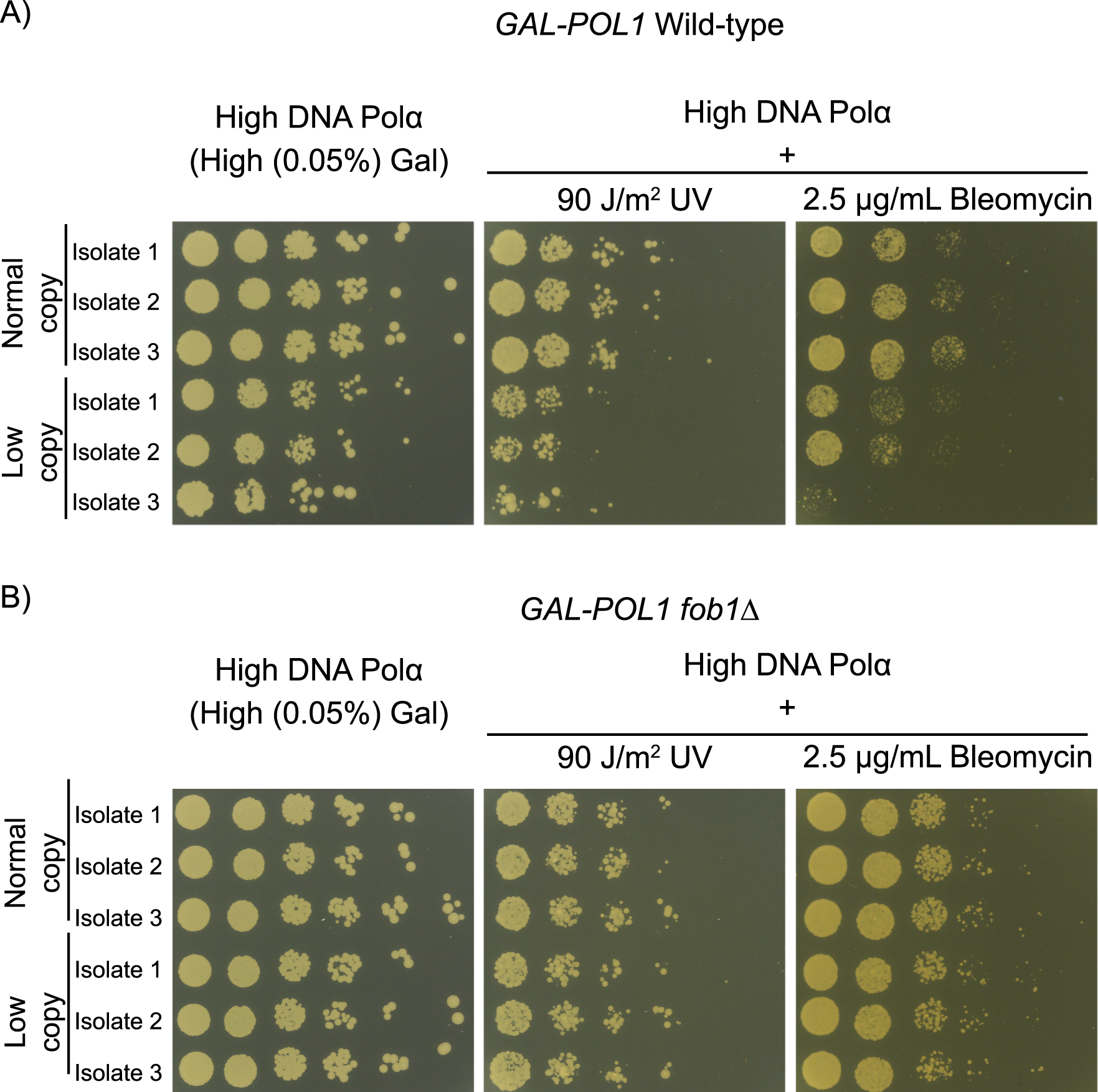

Supplement: S2 Fig — A) Wild-type or B) fob1Δ GAL-POL1 cells were subcultured in medium containing high or low levels of galactose for ~50 generations to generate 3 independent isolates each with normal or low rDNA copy number (S2 Table). 5-fold serial dilutions of these isolates were spotted on to high galactose medium containing bleomycin, or spotted on to high galactose medium followed by irradiation with UV. (TIF) [file pgen.1007006.s002.tif]

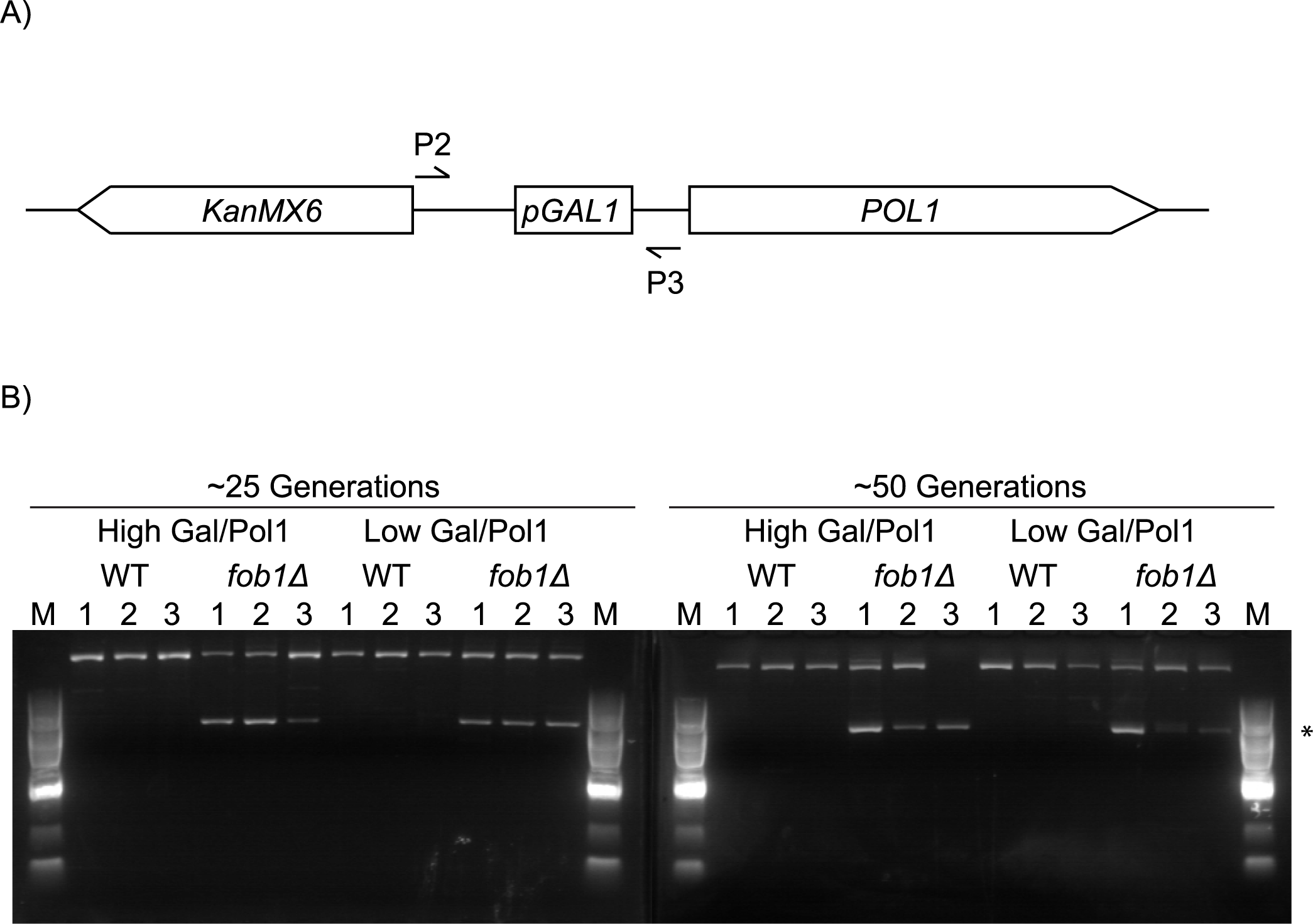

Supplement: S3 Fig — A) PCR scheme and relative positions of primers used [29]. B) Wild-type or fob1Δ GAL-POL1 cells were subcultured in either high/low galactose. After ~50 generations, 3 independent isolates from each condition were used to isolate genomic DNA for PCR. PCR products were run on a 1% agarose gel. M– 100bp marker. *—Non-specific band. (TIF) [file pgen.1007006.s003.tif]

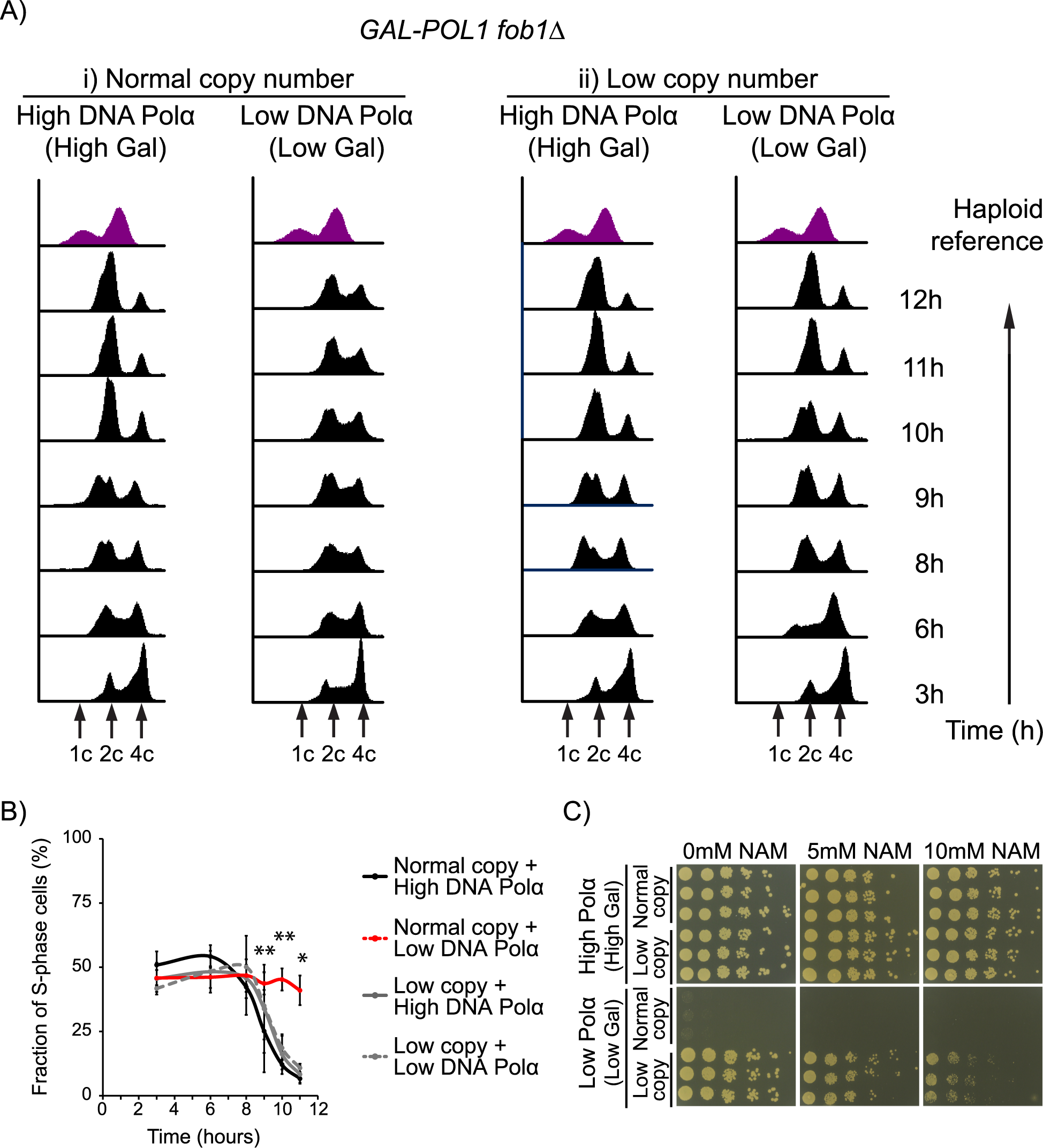

Supplement: S4 Fig — fob1Δ GAL-POL1 cells were subcultured in medium containing high or low levels of galactose for ~50 generations to generate 3 independent isolates each with normal or low rDNA copy number (S2 Table). (A) Representative DNA content profiles over time of asynchronous cultures of fob1Δ isolates with normal (i) and low (ii) rDNA copy number following inoculation into the indicated medium which determines high or low levels of DNA polymerase α. (B) Fraction of cells in S-phase in each of the 4 conditions in (A). Error bars indicate standard deviation based on 3 independent isolates. Statistical significance of differences between fraction of cells in S-phase in high and low levels of DNA polymerase α was calculated using a standard 2-tailed t-test. *—p<0.05, **—p<0.01. (C) Increased rARS firing in nicotinamide exacerbates growth defects under conditions of DNA replication stress. (TIF) [file pgen.1007006.s004.tif]
